# Switch costs reveal vocal-specific executive demands during flexible action selection in crows

**DOI:** 10.1098/rspb.2026.1105

**Published:** 2026-10-14

**Authors:** Diana A. Liao, Felix Kürble, Andreas Nieder

**Affiliations:** Animal Physiology Unit, Institute of Neurobiology, University of Tübingen, Tübingen, Germany

**Keywords:** executive function, vocal-motor control, corvids, volition

## Abstract

Crows and other corvids exhibit remarkable cognitive flexibility, exercising volitional control that allows them to select, initiate and withhold actions based on deliberate decisions rather than reflex. Among these behaviours, vocalizations are especially demanding, yet crows perform them with exceptional precision, paralleling core building blocks of human speech. Despite this, the executive mechanisms supporting intentional vocal control, and how they differ from those governing other voluntary actions, remain largely unknown. We trained carrion crows to produce either vocalizations or pecking responses to arbitrary visual cues presented in randomized, alternating or blocked sequences that controlled how often response modalities switched across trials. While crows performed both actions with high accuracy, vocalizations were consistently slower and more variable than pecking. Notably, vocalizations showed clear switch costs; transitions into vocal responses caused slower reaction times and occasional errors, whereas pecking was largely unaffected. These results show that crows can flexibly switch between motor modalities and that volitional vocalizations carry specific cognitive control demands, previously undescribed in birds. Crows thus stand out as a rare non-primate species in which volitional vocalizations integrate speed, accuracy and executive control.

## 1. Introduction

Volitional control over behaviour is a hallmark of advanced cognition, allowing animals to select, initiate and withhold actions according to context rather than reflex or habit. Such flexibility is critical for adapting to dynamic environments and is observed across taxa, from primates to birds [1]. Corvids, in particular, exhibit remarkable behavioural flexibility, using tools [2–4], planning actions [5–7], generating complex sequences [4,8,9], maintain information in working memory [10–13] and learning from social cues [14–16].

A particularly intriguing capacity of corvids is their ability to exercise volitional control over vocalizations, a specialized and cognitively demanding form of motor action. Carrion crows (*Corvus corone*) can produce or suppress calls in response to arbitrary visual cues, even when cue contingencies were reversed, showing that vocal responding was not driven by any specific salience associated with that stimulus [17]. Crows can even modulate the number of calls in planned sequences [18,19], indicating that vocal output is under cognitive control, paralleling aspects of primate vocal behaviour [20–25].

In humans and non-human primates, volitional control extends to both manual and vocal actions [26–30]. Deliberately switching between different types of motor output—such as hand movements versus vocalizations—requires high-level behavioural flexibility and is commonly measured as switch costs, performance decrements that occur when shifting between response sets compared with repeating the same set [31–33]. These costs are interpreted as reflecting the executive effort needed to deactivate one task set and activate another, offering a window into cognitive control [34,35].

Despite the importance of these processes, the capacity for flexible, intentional control over vocal and non-vocal actions in birds remains poorly understood. To address this, we trained carrion crows on a flexible action-switching task in which arbitrary visual cues signalled either vocalizations or pecking responses. We tested two hypotheses. First, crows can flexibly and reliably switch between cued vocal and pecking actions across sequences. Second, transitions into the more cognitively demanding vocal modality will increase switch costs in reaction time (RT), revealing executive control mechanisms not previously documented in birds. By quantifying these behaviours, we provide a comparative framework for understanding how crows exercise cognitive control across motor domains.

## 2. Methods

### (a) Animals

Two male carrion crows (*C. corone*, 9 years old) participated in these experiments. They were hand-raised and housed socially in large indoor aviaries at the University of Tübingen. Additional details on animal husbandry and housing are described in [36]. Water was available ad libitum during experiments and in the aviary. The crows were on a controlled feeding protocol during training and testing periods. Daily food was provided as rewards during experimental sessions and supplemented afterwards as necessary. All procedures were carried out in accordance to German and European law and were approved by the national authorities (Regiergunspräsidium Tübingen).

Both animals had previous experience with producing both singular and multiple vocalizations and interacting with the touchscreen [17,18,37]. At the beginning of behavioural training, the crows were rewarded with food for producing vocalizations in all contexts (e.g. in the aviary) by the experimenter. After vocalizations were emitted reliably, the behaviour was transferred to the experimental chamber where crows were rewarded for a vocalization using an automated feeder when a blue square was present on the touchscreen. The crows were then trained on a second task where cue colours were modified (Go cue for vocalization is a white square) to test that any response to the blue square was not owing to a special saliency associated with blue [17]. Afterwards, crows were then trained to make not just a single vocalization but a specific number of vocalizations (1 to 4) [18]. The visual cue for a single vocalization was a blue number 1 and the auditory cue, a guitar chord. In this numerical vocal production task, crows needed to peck at the screen (at a ‘confirmation stimulus’) to report that they are done producing the vocal sequence.

Over the years, the crows learned to associate a variety of visual cues with flexible responses including a blue (and later white) square for a single rewarded vocalization, coloured numerals and short sounds for vocal sequences, and pecking responses were shaped from a yellow-cued target to a grey confirmation square. Both crows had comparable prior experience with similar vocal tasks before the present experiment, suggesting that differences in training history are unlikely to account for the pattern reported here.

### (b) Experimental apparatus and design

The crows were trained and tested in a darkened operant conditioning chamber in front of a touchscreen monitor (3M Microtouch, 38.1 cm, 60 Hz refresh rate). Visual stimuli were presented on the monitor using the CORTEX program (National Institute of Mental Health, USA) with a viewing distance of 14 cm. Food rewards—bird pellets (Beo Special, Vitakraft) and mealworms (*Tenebrio molitor*) were provided via an automated rotating feeder after each correctly performed trial. To analyse the vocalizations, a condenser directional microphone (Sennheiser MKE 600) was positioned on top of the monitor leading to a recording computer running a custom-written MATLAB program for vocal detection and to Audacity software for offline analysis of acoustic features. Additional equipment includes a loudspeaker (Vision WB10) to provide acoustic feedback and an infrared camera (Genius iSlim 321R) for experimental monitoring.

To investigate the flexible switching between vocal and pecking responses under varying stimulus sequence designs, we designed an experimental paradigm with differing stimuli for eliciting vocal and pecking responses (figure 1A) which were presented in three different sequences (figure 1B). To initiate a trial, crows position their heads (with an attached reflective foil) centrally 14 cm in front of the touchscreen monitor. This action closes a reflexive infrared light barrier positioned at the top of the experimental box. A response cue then appeared on the touchscreen for 500 ms which indicated the type of response required on that trial; the response cue was a blue square for vocal responses and a yellow circle for peck responses. The response cue then disappeared and was replaced by a grey square during the delay phase of at least 800 ms with an additional random delay of up to 200 ms. Then, the Go cue (a larger lighter grey square) appeared, signalling the crow to make the required response within 15 s. A lack of response within this time was considered a miss. This response period was shortened to 3 s in later analyses of this dataset. If the response type matched the response cue, the trial was correct and the crow got a food reward. If the response type did not match, it was an error and the trial proceeded to a brief time out of 1.8 s. While postural adjustments for the production of each response type might differ, the time to make these postural adjustments would not completely explain the large difference in RTs between response types.

**Figure 1.**
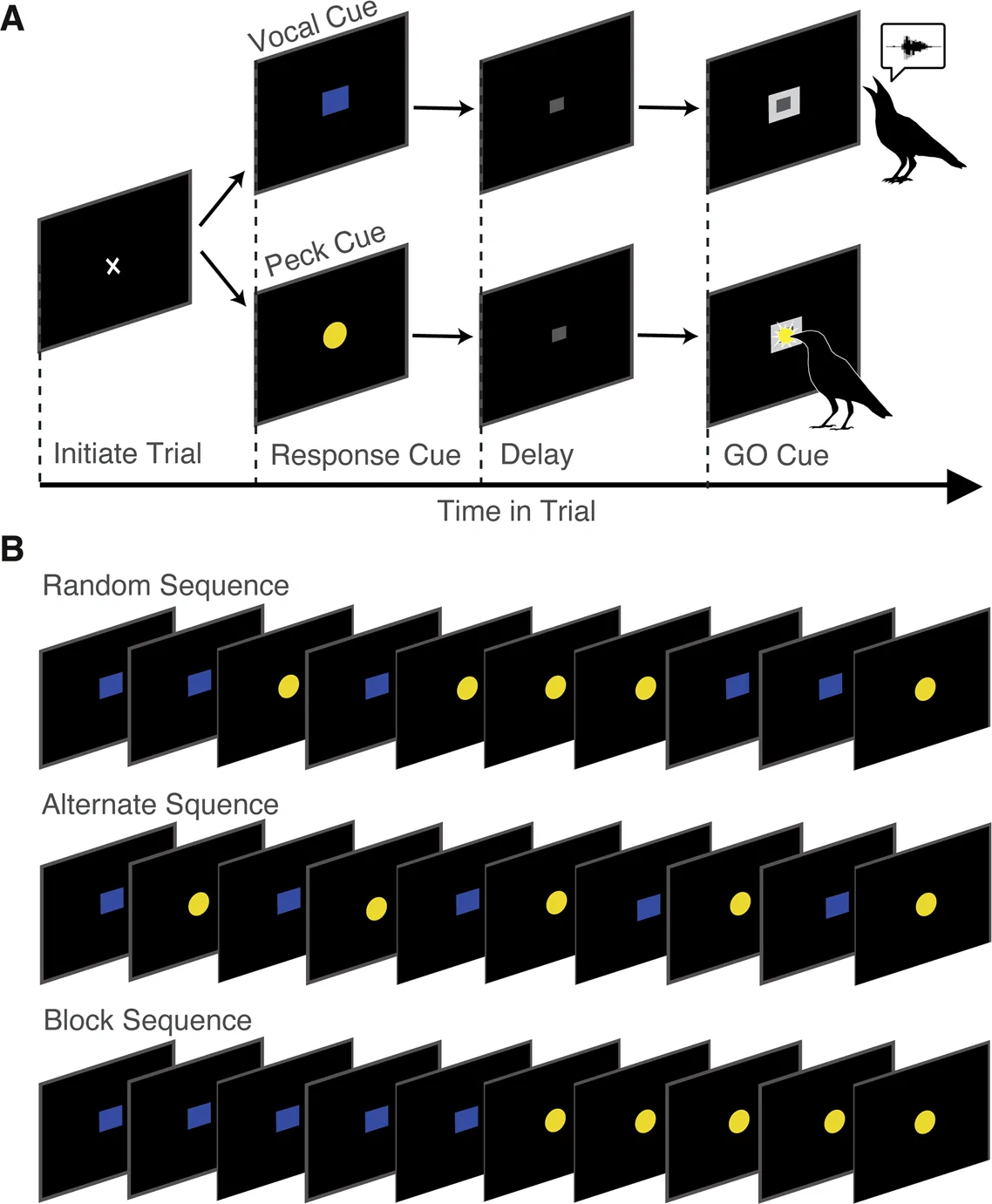
Behavioural paradigm for flexible switching of vocal and pecking responses. (A) After initiating a trial, a response cue appeared informing the crow on the type of motor response to make. Then, the response cue disappeared during the delay phase until the same Go cue appeared signalling the start of the vocal or pecking response. (B) The trials were organized into three different sequences where they were presented in a random, alternating or block order.

The order in which response cues were presented differed in three sequence types:

— *Random* sequence: the response types were presented in a randomized order with a balanced distribution of vocal and pecking responses.
— *Alternating* sequence: response types switched every other trial, so the vocalization and pecking trials alternated in a fixed 1:1 pattern.
— *Block* sequence: the same response type was tested 10 times in a row followed by 10 repetitions of the other response type before switching again (10× voc → 10× peck → 10× voc …).

### (c) Training and data collection

Prior experiments have already established the response cue for vocalization (i.e. blue square) with an immediate vocal response. To introduce the delay and Go cue phases of the task, the Go cue would appear after the response cue. Vocalizations during the response cue were not rewarded and would trigger the end of that trial. After consistent responding during the Go cue, a short delay of 100 ms between the cues was added. This delay was gradually increased by 100 ms after a set number of 60 to 120 successful trials until it reached the required timing. This process was repeated for the response cue associated with pecking.

To better establish that the response cues were associated with a required motor response, they were combined during a training session in large block sizes of 30 consecutive trials per response type. Over days, we then gradually reduced the block size to two consecutive trials. After the crows could perform above 70% accuracy over at least 120 trials, we began the data collection phase with the three different sequence types.

Each sequence type (i.e. *random*, *alternating* and *block*) was recorded for 10 days each for a total of 30 days of recordings per crow. Sequence order was counterbalanced across six-day blocks, yielding two complete cycles through all three sequence types. As the crows occasionally showed extended pauses in responding, a recording session was only included in the final analysis if at least 150 trials were performed within a 1-hour period. The experiments in this study were conducted between April and July 2025.

### (d) Vocalization processing

A custom MATLAB routine automatically detected the onset and offset of any acoustic signal that differed from the background noise. The boundaries of each trial were identified using numbered strobes sent from the CORTEX system while running the behavioural task. A quiet segment at the start of the experimental session each day was used as the baseline noise level. The envelop of the sound amplitude waveform was thresholded and consecutive segments longer than 50 ms were flagged as potential vocalizations. Onset and offset times were saved and later manually verified.

### (e) Behavioural and statistical analysis

Performance and RTs were collected during the experiment and analysed offline using MATLAB v. 2020b (Mathworks). The RT was calculated as the onset of the response cue and the onset of the vocalization or the peck. For comparison with previous publications, we cut off the time to respond to 3 s, reclassifying 4% of trials as correct responses to misses. These reclassified trials fell on the long tail of the response time histograms. All main analyses are shown with the original 15 s window compared to the shortened 3 s window in table S1 in the supplementary material.

We compared the percentage of hits, errors, misses between vocalization and peck trials. We further compared differences in the percentages for different sequence types. To test for significant differences, we fit a linear mixed-effects model (function: lmefit) with hit rate as the dependent variable and modality (vocalization versus peck) as a fixed effect. Random intercepts were included for BirdID (crow 1 versus crow 2) and for session nested within BirdID to account for individual differences and session-to-session variability.

The linear mixed-effects model to test for effects of response modality on hits was:

hits∼modality+(1|BirdID)+(1|BirdID:Session)

In this model, the degrees of freedom (d.f. = 118) equal the number of observations minus the number of fixed-effect parameters. We repeated similar models using error rate and miss rate as the dependent variables. We report the *β* value, *t*-statistic and *p*-value.

To examine the effect if sequence type, we fit similar models as above using sequence type (*random*, *alternating* and *block*) as the fixed effect alongside BirdID and Session nested within BirdID for each modality separately.

The linear mixed effects model to test for effects of sequence type on hits was:

hits∼sequence+(1|BirdID)+(1|BirdID:Session)

We used type III analysis of variance to test the overall effect of sequence, reporting the *F*-statistic and *p*-value. The numerator degrees of freedom equal to 2 (3 sequence types -1) and the denominator degrees of freedom was calculated using the residual method. We ran similar models using error rate and miss rate as the dependent variables.

To complement session-level analyses, we also aggregated performance across all trials to examine differences between response modalities and sequence types (supplementary material, figure S1). We calculated proportions by taking the correct number of responses for vocalizations and peck trials and dividing by the total number of those trials. We ran chi-squared tests for the difference in proportion between vocalization and pecking, reporting the chi-square statistic and the *p*-value. For trial-level differences in sequence types, we followed up significant omnibus chi-square tests with pairwise chi-square tests. To correct for multiple comparisons, we perform a Bonferroni correction (*α* = 0.017 for three comparisons).

Changes in RTs were examined by taking the median RT for each response modality for each session. To examine differences in RT by response modality and sequence type, similar LME models as above were fit to these session medians. Additionally, we calculated the variability in RT across trials for each session by taking the standard deviation of all the RTs (supplementary material, figure S2). We use LME models to examine differences by response modality and sequence type.

To examine switch costs, performance decrements that occur when shifting between response sets compared with repeating the same set, we calculated the RT for vocalization trials when they were preceded by vocalization trials (same) or peck trials (switch). Similarly, we calculate peck RTs following a previous peck or vocal response. Given that the *alternating* sequence only consisted of switches, we focused our analysis on *random* and *block* sequences. We take the median of each session for a total of 10 paired values for each sequence type per crow. Because RTs are not-normally distributed, we compared the same and switch values for each crow separately and together using Wilcoxon signed-rank tests. We report the *W* statistic and *p*-value.

To examine the dynamics surrounding switch trials in *block* sequences, we find the sequences of correct 20 trials surrounding a switch from pecking (10 trials) to vocalization (10 trials). We calculate the difference in RT between the first vocalization trial after the task response modality (Voc 1) and the mean of all subsequent vocalization trials (Voc 2-10). We compared the two RT distributions with a paired *t*-test. We report the *t* statistic and *p*-value. We repeat these tests for comparing the first vocalization trial RT (Voc 1) with the second vocalization trial RT (Voc 2) and for comparing the second trial RT (Voc 2) with the third trial RT (Voc 3).

## 3. Results

We trained two carrion crows to vocalize or peck the screen in response to the presentation of arbitrary visual cues to receive a reward (figure 1A). Vocalization trials and peck trials were presented in three different sequences over days: *random* (vocalizations and pecks cued in random order), *alternating* (vocalization and pecks alternating every trial) and *block* (10 consecutive trials of one response type before switching to 10 consecutive trials of the other and repeating this pattern) (figure 1B). We collected data from 30 daily sessions from each crow with each day assigned to a particular sequence in randomized order resulting in 10 sessions per sequence type. These different sequences were included to test whether switching between response modalities and the predictability of those switches affects performance. We predicted that the *alternating* sequence would be more difficult than the *block* sequence, owing to the higher number of switches. Additionally, we expected that the *random* sequence would be the most challenging as switches could not be anticipated whereas for both the *alternating* and *block* sequences*,* responses could potentially be prepared.

Representative performance of a single session (*random* sequence, session no. 26) from crow 1 is shown in figure 2, with vocalization trials presented in figure 2A and peck trials in figure 2B. The crow produced each response type reliably and almost exclusively to the corresponding visual cue. Pecking responses were rapid and nearly error-free, whereas vocalization responses exhibited longer RTs with occasional errors and misses.

**Figure 2.**
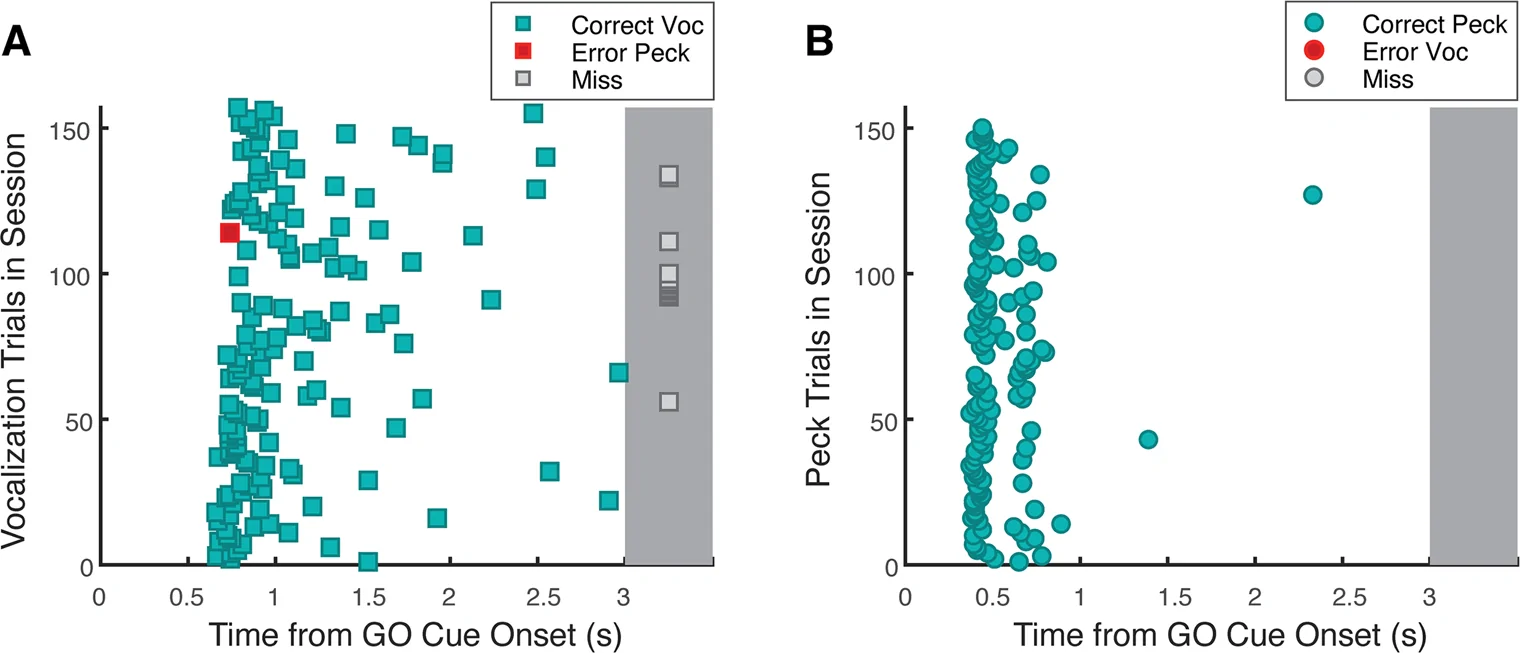
Example session performance for vocal and peck trials. All responses for crow 1 in a *random* sequence session, split into (A) vocal and (B) peck trials sorted by time in session. Turquoise markers indicate the response time after Go cue onset of correct response (square: vocalization, circle: peck). Red markers indicate errors, and grey markers indicate misses occurring 3 s after Go cue onset.

### (a) Performance accuracy

This pattern reflected the average performance of both crows: overall accuracy across the three different response sequences was high, but hit rates—calculated as the number of correct trials over the total number of presented trials for that modality—were slightly lower for vocalization trials (92.2%) than for peck trials (98.2%; figure 3A). This hit rate difference was significant (linear mixed-effects model: *β* = 0.06, *t*(118) = 8.32, *p* < 0.0001). Error rates were very low for both response types (vocalization: 2.9%; peck: 1.3%; figure 3B) and differed significantly between response modalities (*β* = –0.02, *t*(118) = –2.82, *p* = 0.0057). Lastly, miss rates were also low for both response types (vocalization: 4.9%; peck: 0.5%; figure 3C) but higher for vocalizations (*β* = –0.04, *t*(118) = –7.77, *p* < 0.0001).

**Figure 3.**
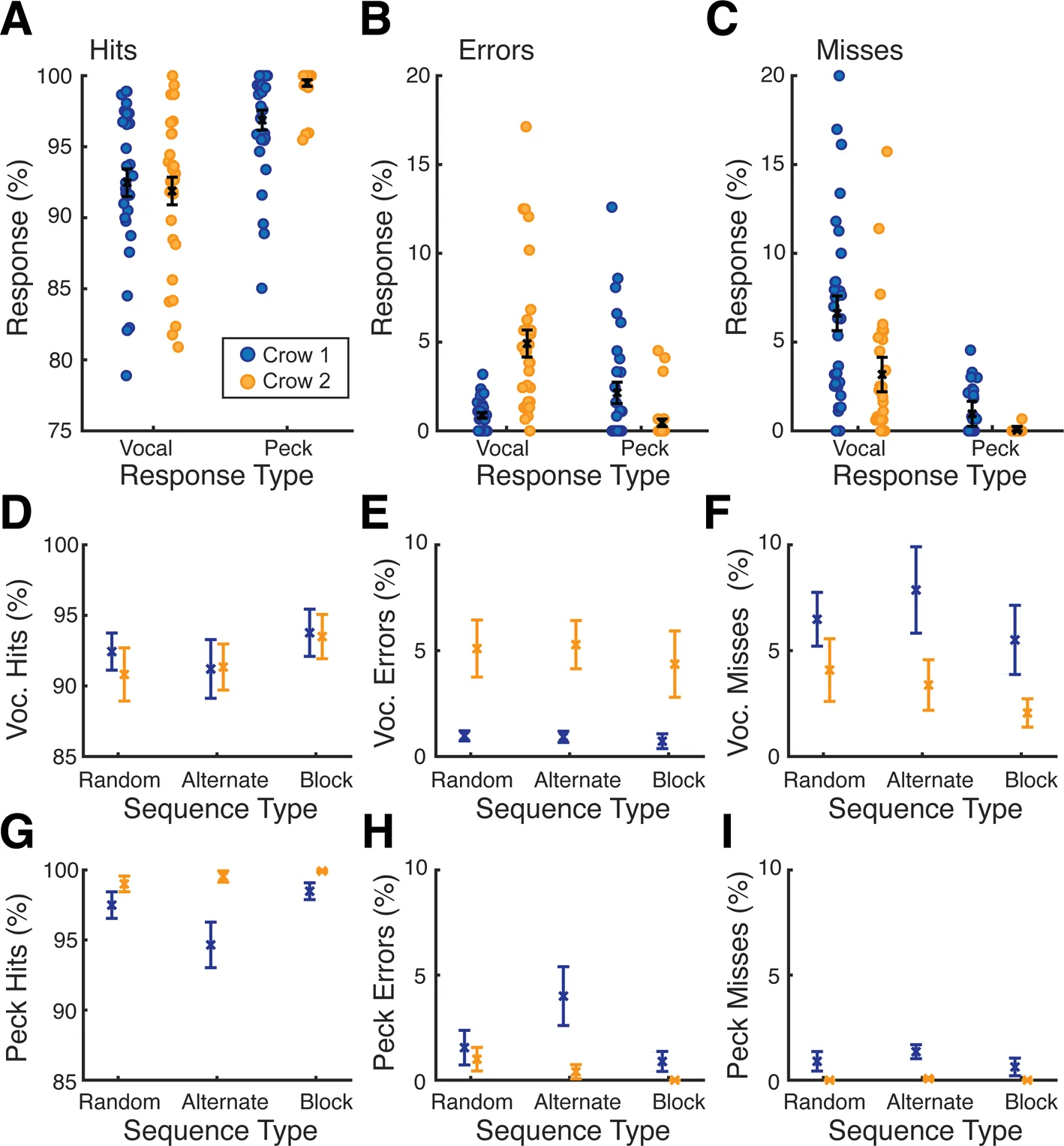
Vocal and pecking performance. (A–C) Performance between response modalities. (A) Percent of hits (correct responses) are plotted for vocal and peck responses. Colours indicate crow identity. Individual coloured dots represent sessions and black error bars are the mean and standard error of all sessions. Same for (B) errors and (C) misses. (D–F) Vocal performance for the three different sequence types, displayed for (D) hits, (E) errors and (F) misses. Error bars indicate the standard error of the mean (SEM) over 10 session for each sequence type individually for each crow. (G–I) Peck performance for the three different sequence types, displayed for (G) hits, (H) errors and (I) misses. Error bars as in above.

Vocal performance was high for all three response sequence types individually, including the random sequence (*random*: 91.6%; *alternating*: 91.3%; *block*: 93.6%; figure 3D). Performance did not differ across sequence types (*F*(2, 57) = 1.22, *p* = 0.30). Performance was higher for pecking responses (*random*: 98.3%; *alternating*: 97.1%; *block*: 99.2%; figure 3G) and also did not differ across sequence types (*F*(2, 57) = 2.99, *p* = 0.06). Similarly, error rates did not differ across sequence types for vocalization trials (*F*(2, 57) = 0.21, *p* = 0.81; figure 3E) or peck trials (*F*(2, 57) = 2.76, *p* = 0.07; figure 3H). Miss rates did not differ across sequence types for either response modality (vocalization: *F*(2, 57) = 0.98, *p* = 0.38; peck: *F*(2, 57) = 1.00, *p* = 0.37; figure 3F,I). Together, these results demonstrate that performance is robust to variations in sequence structure, maintaining high accuracy regardless of whether cues were randomly interleaved, alternated or presented in short blocks.

Complementing these session-level analyses, we collapsed across all trials (supplementary material, figure S1) and found a significant difference in hit rates between vocalizations and pecking (crow 1: $x^{2}(1)=\;$69.6, *p* < 0.0001, crow 2: $x^{2}\;(1)=293.8$, *p* < 0.0001). Also collapsing across trials, we find no significant difference between sequence types for vocalization (crow 1: $x^{2}(2)=3.53$, *p* = 0.17, crow 2: $x^{2}(2)=5.11$, *p* = 0.08). However, we did find a significant difference between sequence types for pecking (crow 1: $x^{2}(2)=31.70$, *p* < 0.0001, crow 2: $x^{2}(2)=14.23$, *p* = 0.0008). Follow-up pairwise tests revealed that *random* and *block* sequences had higher performance compared to *alternating* sequences for crow 1 and the *block* sequences had higher performances compared to *random* sequences for crow 2.

### (b) Response times

RTs mirrored the differences between response modalities. Median RTs for vocalizations were 0.85 s for crow 1 (figure 4A) and 1.53 s for crow 2 (figure 4B). Median RTs for pecks were faster at 0.46 s and 0.54 s for crow 1 and crow 2, respectively (figure 4A,B). Thus, vocalization RTs were consistently slower than peck RTs. This pattern was confirmed when comparing session medians for RTs (figure 4C) where RTs showed a significant main effect of response modality (*β* = –0.69, *t*(118) = *–*15.19, *p* < 0.0001).

**Figure 4.**
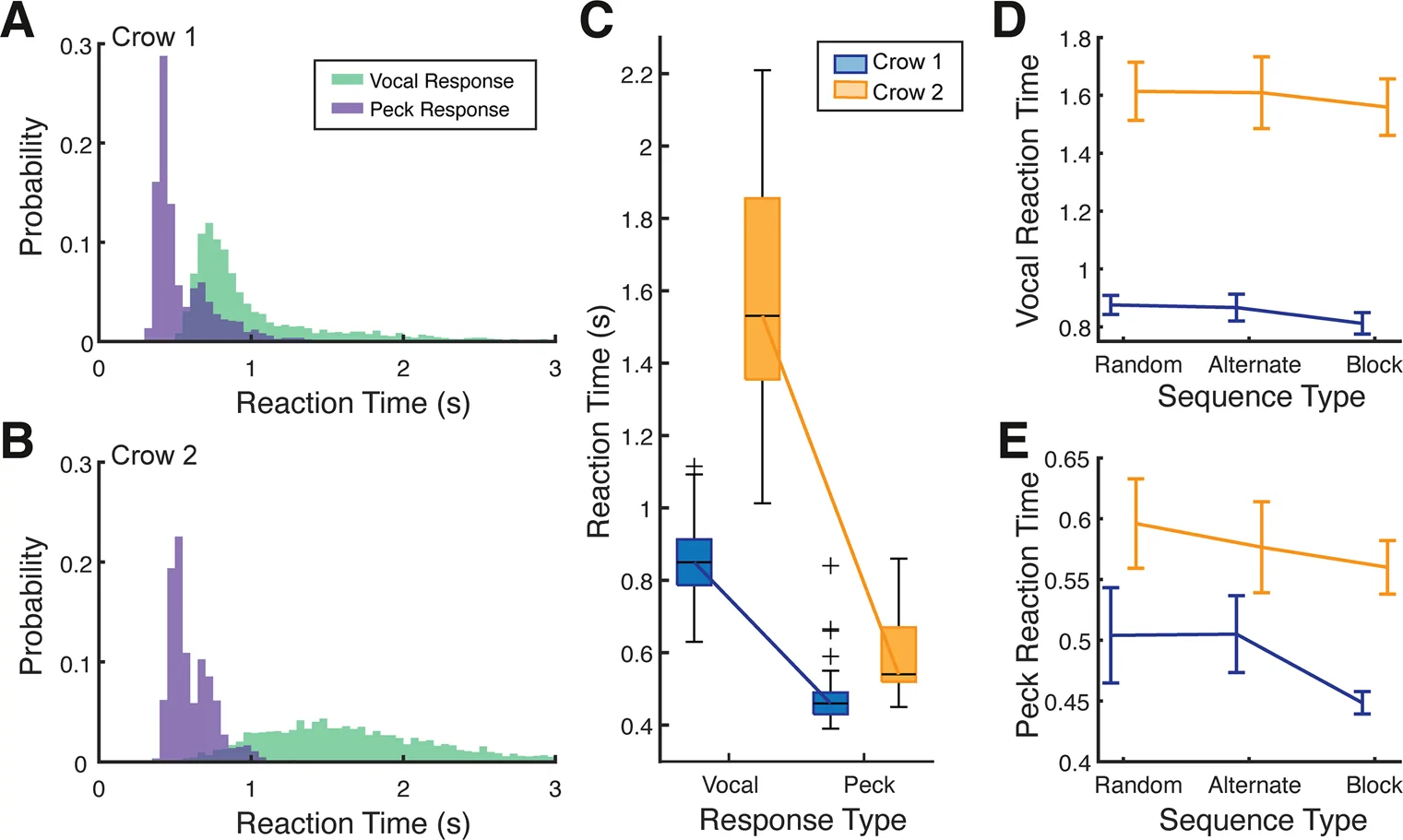
RT differences for vocal and pecking responses. (A) Histogram of RTs for vocal responses (green) and pecking responses (purple) for crow 1. (B) RT histograms for crow 2. (C) Box plot showing differences in median RTs for different response types in each crow. (D) RT for vocal responses separated out by sequence type. (E) RT for pecking responses separated out by sequence type. Colours indicate crow identity. Error bars indicate the SEM.

When split by sequence type, *block* trials showed the fastest RTs for both vocalization and peck responses (figure 4D,E). For crow 1, median vocalization RTs were 0.88 s (*random*), 0.87 s (*alternating*) and 0.81 s (*block*), while peck RTs were 0.50 s, 0.51 s and 0.45 s, respectively. Crow 2 exhibited slower RTs overall, with vocalization RTs of 1.61 s (*random*), 1.61 s (*alternating*) and 1.56 s (*block*), and peck RTs of 0.60 s, 0.58 s and 0.56 s. Linear mixed-effects models revealed no significant effect of sequence type (vocalization: *F*(2, 57) = 0.34, *p* = 0.71; peck: *F*(2, 57) = 1.28, *p* = 0.29).

Additionally, to examine the variability in RTs across trials for each session, we calculated the differences in the standard deviation of the RTs between response modalities (supplementary material, figure S2). We found that vocalization RTs were more variable than peck RTs (*β* = –0.25, *t*(118) = –15.34, *p* < 0.0001). However, there was no significant effect of sequence type on trial RT variability for both response modalities (vocalization: *F*(2, 57) = 0.30, *p* = 0.74; peck: *F*(2, 57) = 0.67, *p* = 0.52).

### (c) Switch costs between response types

The slightly higher accuracy and faster RTs in *block* sequences suggest potential switch costs in the *random* and *alternating* sequences that are reduced in the *block* sequences. To examine these putative switch costs, we analysed transitions between different response types (vocalization → peck, peck → vocalization) and compared them to transitions within the same response type (vocalization → vocalization, peck → peck). Analyses were conducted separately for *Random* and *Block* sequences and for each bird (figure 5A–D).

**Figure 5.**
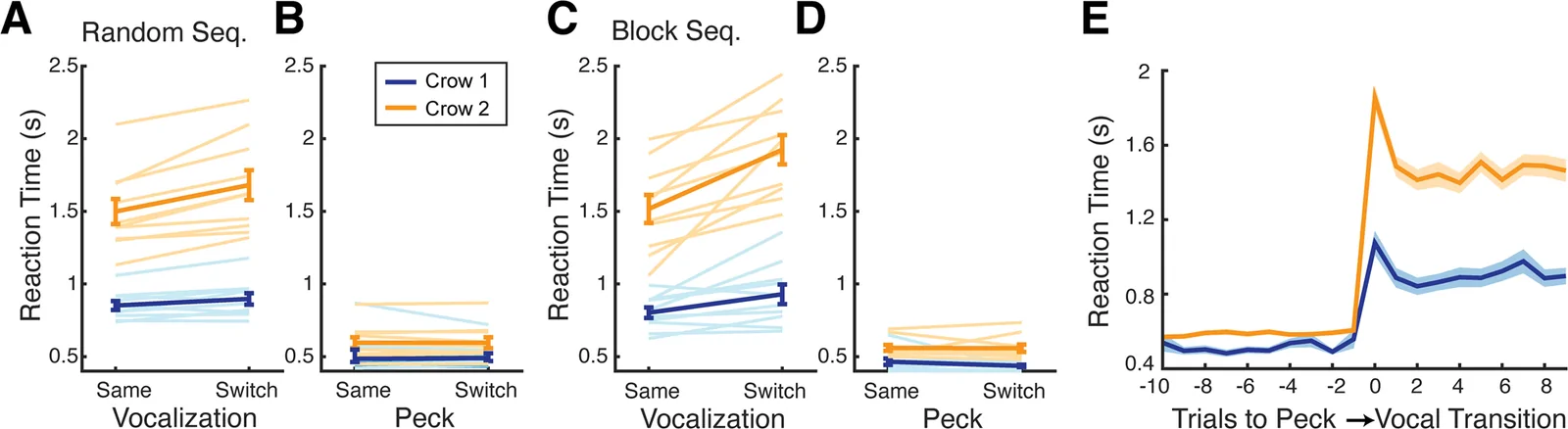
Switch costs between response types. (A) RTs for vocalizations that follow a previous vocalization trial (same) or a peck trial (switch) are shown for *random* sequence sessions. The lighter transparent lines represent the session medians and the darker, solid lines are the overall mean over sessions and standard error. Colours indicate crow identity. (B) RT for pecks that follow a previous peck trial (same) or a vocalization trial (switch). Similar plots are shown for *block* sequence sessions separating (C) vocalization and (D) peck trials. (E) The RTs of the transition between 10 correct peck trials and 10 correct vocalization trials in *block* sessions are plotted. The shaded error bars indicate the SEM across instances of this 20-trial pattern.

In the *Random* sequence (figure 5A), vocalization trials showed a significant difference between same-type and switch transitions for both crow 1 (*W* = 8, *p* = 0.0488), crow 2 (*W* = 0, *p* = 0.0020) and both crows together (*W* = 9, *p* = 0.0003). This effect was not observed for peck responses (figure 5B; crow 1: *W* = 22, *p* = 0.60; crow 2: *W* = 18, *p* = 1; both crows: *W* = 77, *p* = 0.71).

In *Block* sequences, vocalization trials again showed a significant difference between same-type and switch transitions (figure 5C; crow 1: *w* = 7, *p* = 0.0371; crow 2: *w* = 0, *p* = 0.0020; both crows: *w* = 7, *p* = 0.0003), whereas peck trials did not (figure 5D; crow 1: *w* = 28, *p* = 0.20; crow 2: *w* = 21, *p* = 0.74; both crows: *w* = 92.5, *p* = 0.20). To examine the dynamics of switching from peck to vocal responses, we evaluated the RTs of the 10 trials before and after each transition in *block* sequences. This revealed that the first trial following a switch exhibited a notably slower RT compared to the other trials in the vocal block (figure 5E**;** paired *t*-test, crow 1: *t*(67) = 3.00, *p* = 0.0037, crow 2: *t*(72) = 6.72, *p* < 0.0001). To further examine the transition trajectory into the vocal block, we find that the first vocal trial is significantly slower than the second trial (paired *t*-test, crow 1: *t*(67) = 2.54, *p* = 0.01, crow 2: *t*(72) = 5.36, *p* < 0.0001). The second trial does not differ from the third trial (paired *t*-test, crow 1: *t*(67) = 0.68, *p* = 0.50, crow 2: *t*(72) = 1.10, *p* = 0.28).

## 4. Discussion

### (a) Advanced vocal flexibility in corvids

Our experiments show that crows exhibit remarkable flexibility in switching between response types, as evidenced by their high performance even in random sequence sessions. Although both response modalities were performed successfully, volitional vocalizations were more demanding than volitional pecking, as reflected in lower accuracy and slower RTs. Compared to head and beak movements—which likely reflect an ancestral trait in theropods such as birds—vocal production requires tightly coordinated control over multiple interacting systems, including respiration, laryngeal or syrinx activity and oral articulation (tongue, jaw and lips/beak) [38–40]. From a cognitive perspective, this places greater demands on planning, sequencing and feedback monitoring.

Our observations align with previous work demonstrating flexible vocal control in corvids: trained carrion crows can volitionally emit and withhold calls in response to arbitrary visual cues, showing that vocalizations are not purely reflexive but under cognitive control [17]. In these tasks, crows rapidly learned to vocalize to ‘Go’ cues and to suppress vocalizations to ‘No-Go’ cues, and could relearn reversed cue meanings, demonstrating rapid remapping of vocal responses to neutral cues. Other work demonstrated that crows can modulate the number of vocalizations, flexibly producing sequences of variable call numbers in response to arbitrary cues, indicative of planned vocal output and control processes [18]. Exploratory studies in rooks (another corvid) show spontaneous coordination of vocalizations with external events, further suggesting contingent vocal control in naturalistic contexts [41]. Together with evidence of vocal learning and mimicry across many corvid species [42–45], these findings underscore advanced control over vocal output in corvids that parallels aspects of human and non-human primate volitional vocal behaviour [21,23,24,30,46,47].

### (b) Volitional motor actions in birds and primates

The performance of our crows is the first evidence of a non-human species that can flexibly and robustly switch between vocal and non-vocal response modalities on a trial-by-trial basis. Experiments involving response switching in macaque monkeys have either used different individuals for each response modality [29] or have longer block sequences with low switching frequency [26].

We directly compared the performance of our crows to two rhesus macaques performing a similar protocol to produce volitional calls and hand movements [26], revealing both parallels and differences in response dynamics. These cross-species comparisons should be interpreted in light of differences in training history, task implementation and response modalities, which may influence absolute RTs and accuracy. At the same time, the present datasets offer a rare opportunity for comparison across evolutionarily distant species tested within closely aligned task structures and analytical frameworks. Accordingly, our focus is not on establishing strict quantitative equivalence between species, but on evaluating whether comparable experimental conditions reveal similar or divergent qualitative patterns of performance and control.

For vocalizations, crows were faster and more accurate than macaques: crow 1 and crow 2 had median RTs of 0.85 and 1.57 s, with hit rates of 92.5 and 91.9%. By contrast, monkey A and monkey P were slower (2.00 and 1.64 s) and less accurate (38% and 55%) [26]. These comparisons highlight that crows produce vocal responses more rapidly and reliably, potentially reflecting specialized neural adaptations for learned vocalizations [48–51]. Intriguingly, humans have also evolved specialized adaptations for speech production, including expanded cortical representation of the larynx, direct cortical control of vocal motor neurons and recruitment of additional brain areas [22,52–54]. Comparative studies across species with convergently evolved vocal abilities can reveal both universally shared and lineage-specific divergent solutions for complex motor control.

By contrast, for non-vocal motor responses—manual movements and pecking—two functionally similar actions for manipulating objects [4,55], performance was comparable across species. Crows (0.46 and 0.55 s) and monkeys (0.51 and 0.54 s) showed quick response times and high accuracy [26], suggesting that birds and primates demonstrate similar proficiency in non-vocal goal-directed actions [56–58].

Overall, both crows and monkeys perform non-vocal actions more efficiently than vocal actions [26], suggesting that vocalization might require more time for motor preparation compared to manual actions [29]. However, crows demonstrate greater cognitive control over vocal output by combining speed and accuracy in volitional vocalizations in a way that non-human primates do not appear to.

### (c) Executive control underlies vocal switch costs

A hallmark of executive control in humans and other animals is the switch cost—the performance decrement observed when changing between response sets relative to repeating the same set [31]. Switch costs are widely interpreted as reflecting the time and effort required to deactivate a previous task set and activate a new one, a core signature of executive control. In our crows, switch costs were asymmetric and modality-specific. Vocal responses showed clear performance costs when switching, whereas pecking responses were largely unaffected. This suggests that initiating a deliberate vocalization requires greater cognitive reconfiguration than pecking, resulting in switch costs.

By contrast, most bird studies fail to show classic switch costs. In pigeons, switching among categorization tasks generally does not affect accuracy or RT, even when trial structures resemble human paradigms. This implies that associative learning, rather than task-set reconfiguration, underlies their flexible performance [59,60]. The asymmetric vocal switch costs observed in crows therefore stand out, suggesting that, in corvids, such costs may reflect executive control processes more akin to those reported in primates [61].

Evidence from non-human primates is mixed. Early studies in rhesus macaques reported relatively low switch costs but robust interference effects in arbitrary task-switching paradigms, contrasting with the larger switch costs typically seen in humans [62]. More recent studies have found clear switch costs in macaques under paradigms more closely aligned with human protocols [63]. Across species, the magnitude and expression of switch costs depend on task demands, training and cognitive strategies.

Theoretical accounts of switch-cost asymmetries differ in whether they emphasize inhibitory control, interference between competing task sets or differences in reconfiguration demands across tasks ([34,64–66],). One interesting finding across several task-switching studies is that switching to a dominant (generally better trained, putatively easier) task results in larger switch costs compared to switching to a less dominant (putatively harder) task [67,68], suggesting asymmetrical inhibitory processes. Critically, switch-cost asymmetries did not only rely on more or less dominant task representations, but can also be affected by the response modalities to tasks [64]. In a study of switching between vocal and manual responses, switching to the vocal response was associated with greater performance costs than switching to the manual response [33] (though see [32] for switch costs between manual, vocal and oculomotor response modalities with no significant asymmetry). Taken together, these findings suggest that switch-cost asymmetries are not solely determined by relative task difficulty in a global sense, but may reflect differences in control demands associated with specific response effectors and their associated task sets. In this context, our results indicate that the switch costs observed in crows’ vocal responses may reflect asymmetric executive control demands linked to the recruitment of different response modalities.

## Supplementary Material



## Data Availability

Data and code to reproduce analyses and figures are available at Figshare [69]. Supplementary material is available online [70].
