# Supplementary material for: Switch costs reveal vocal-specific executive demands during flexible action selection in crows
Source: Proc Biol Sci. 2026 Oct 14;293(2080):20261105. doi: 10.1098/rspb.2026.1105 (PMC13678624; doi:10.1098/rspb.2026.1105)
Supplement: Supplementary file 1 [file rspb.2026.1105.zip › rspb.2026.1105/rspb20261105_si_001.pdf]

SUPPLEMENTARY MATERIALS

Switch costs reveal vocal-specific executive demands during flexible action selection in crows

Liao, D.A.*, Kürble, F., & Nieder, A.*

Animal Physiology, Institute of Neurobiology, University of Tübingen, Auf der Morgenstelle 28, 72076 Tübingen, Germany

*Corresponding authors:

Diana A. Liao – diana.a.liao@gmail.com,

Andreas Nieder - andreas.nieder@uni-tuebingen.de; ORCID: 0000-0001-6381-0375

Doi: 10.1098/rspb

Supplementary Table 1: Comparison of analysis performed with 15 second versus 3 second response time window.

Variable of Interest	< 3 sec		< 15 sec	
Hit Rates for Vocalization	92.2%		96.4%	
Hit Rates for Pecking	98.2%		98.6%	
Effect of Response Modality	$\beta = 0.06$, $t(118) = 8.32$, $p< 0.0001$		$\beta = 0.02$, $t(118) = 3.73$, $p = .0003$	
Error Rates for Vocalization	2.9%		2.9%	
Error Rates for Pecking	1.3%		1.3%	
Effect of Response Modality	$\beta =-0.02$, $t(118) = -2.82$, $p = .0057$		$\beta =-0.02$, $t(118) = -2.82$, $p = .0057$	
Miss Rates for Vocalization	4.9%		0.7%	
Miss Rates for Pecking	0.5%		0.1%	
Effect of Response Modality	$\beta = -0.04$, $t(118) = -7.77$, $p<0.0001$		$\beta = -0.005$, $t(118) = -3.92$, $p = .0001$	
Vocal Hit Rate by Sequence Type	$F(2, 57) = 1.22$, $p = 0.302$		$F(2, 57) = 0.30$, $p = 0.740$	
Peck Hit Rate by Sequence Type	$F(2, 57) = 2.99$, $p = 0.058$		$F(2, 57) = 2.77$, $p = 0.071$	
Vocal Error Rate by Sequence Type	$F(2, 57) = 0.21$, $p = 0.813$		$F(2, 57) = 0.21$, $p = 0.813$	
Peck Error Rate by Sequence Type	$F(2,57) = 2.76$, $p = 0.072$		$F(2, 57) = 2.76$, $p = 0.072$	
Vocal Miss Rate by Sequence Type	$F(2, 57) = 0.98$, $p = 0.382$		$F(2, 57) = 0.20$, $p = 0.817$	
Peck Miss Rate by Sequence Type	$F(2, 57) = 1.00$, $p = 0.373$		$F(2, 57) = 1.56$, $p = 0.220$	
Median RT for Vocalization	Crow 1: 0.85s	Crow 2: 1.53s	Crow 1: 0.88s	Crow 2: 1.55s
Median RT for Pecking	Crow 1: 0.46s	Crow 2: 0.54s	Crow 1: 0.46s	Crow 2: .54s
RT effect of Response Modality	$\beta = -0.69$, $t(118) = -15.19$, $p < .0001$		$\beta = -0.72$, $t(118) = -15.05$, $p < .0001$	
RT effect of Sequence Type for Vocalizations	$F(2, 57) = 0.341$, $p = 0.712$		$F(2, 57) = 0.405$, $p = 0.669$	
RT effect of Sequence Type for Pecking	$F(2, 57) = 1.279$, $p = 0.286$		$F(2, 57) = 1.217$, $p = 0.304$	

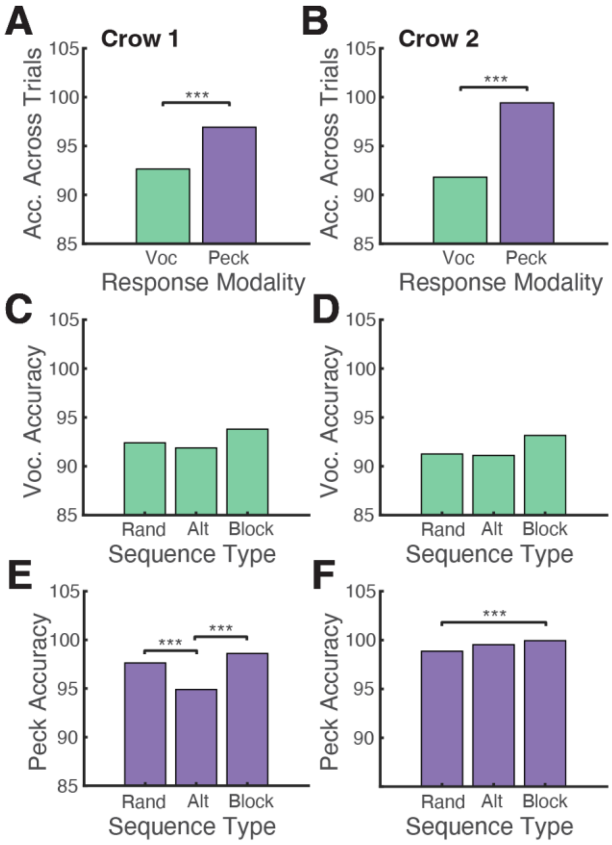

Supplementary Figure 1: Accuracy aggregated across all trials for response modality and sequence types. **(A)** Performance of Crow 1 for all vocalization and peck trials collapsed together.

(B) Same as **(A)** for Crow 2.

(C-D) Performance for the three different sequence types collapsed across all vocal trials for Crow 1 **(C)** and Crow 2 **(D)**.

(E-F) Performance for the three different sequence types collapsed across all peck trials for Crow 1 **(E)** and Crow 2 **(F)**. Significant chi-square tests marked with asterisks.

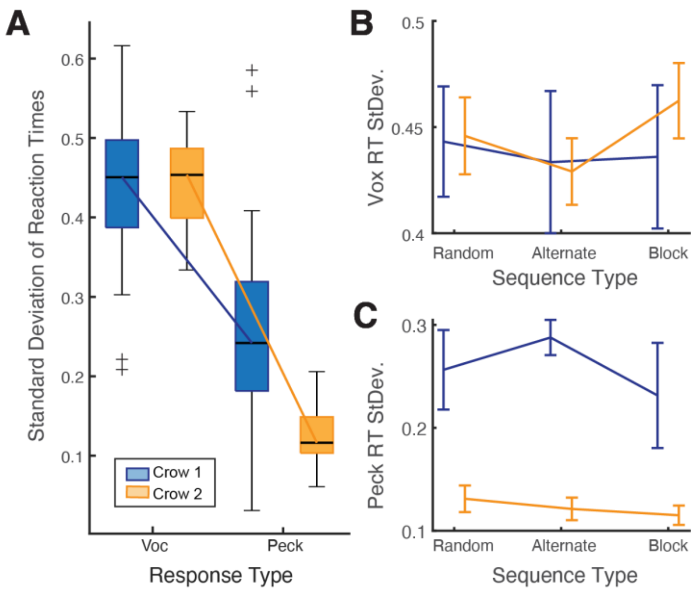

Supplementary Figure 2: Variability in Reaction times across response modality and sequence types.

(A) Box plot showing differences in session standard deviation of reaction times for different response types in each crow.

(B) Standard deviation in vocalization RTs separated out by sequence type.

(C) Standard deviation in peck RTs separated out by sequence type. Colors indicate crow identity. Error bars indicate the standard error of the mean (SEM).
